# Impact of Prolonged Catheterization for Vesicourethral Leakage on Continence and Quality of Life After Robotic Prostatectomy

**DOI:** 10.7759/cureus.99332

**Published:** 2025-12-15

**Authors:** Hisanori Taniguchi, Junichi Ikeda, Monta Inoue, Yuki Masuo, Hidefumi Kinoshita

**Affiliations:** 1 Urology and Andrology, Kansai Medical University, Hirakata, JPN

**Keywords:** robot-assisted radical prostatectomy, surgical robotics, urinary anastomotic leak (ual), urinary catheters, urinary incontinence (ui)

## Abstract

Introduction: To determine whether prolonged urinary catheterization due to vesicourethral anastomotic leakage (VAL) after robot-assisted radical prostatectomy (RARP) impacts urinary continence recovery and quality of life (QOL).

Methods: We retrospectively analyzed 360 patients who underwent transperitoneal RARP at a single institution from January 2022 to December 2024. All patients underwent cystography on postoperative day 6 or 7. Those with VAL requiring extended catheterization were compared with patients undergoing standard removal. Continence was assessed using Question 5 of the Expanded Prostate Cancer Index Composite (EPIC), and QOL was measured with the Functional Assessment of Cancer Therapy-General (FACT-G), FACT-Prostate (FACT-P), and the EPIC urinary subscale. Outcomes were evaluated up to 24 months postoperatively.

Results: VAL was detected in 36 patients (10.0%). Continence recovery did not differ significantly between the prolonged and standard catheter groups at 1, 6, or 12 months (63%, 89%, 92% vs. 54%, 85%, 93%, respectively). QOL outcomes were also comparable, although preoperative EPIC scores were lower in the prolonged catheter group. Multivariate analysis identified greater prostate specimen weight (p = 0.049) and intraoperative leakage (p = 0.002) as independent predictors of prolonged catheterization.

Conclusions: Prolonged urinary catheterization due to VAL after RARP does not adversely affect long-term urinary continence recovery or urinary-related QOL.

## Introduction

Compared with open or conventional laparoscopic prostatectomy, robot-assisted laparoscopic prostatectomy (RARP) offers the advantage of multi-directional instrument mobility, facilitating vesicourethral anastomosis. However, even in the era of minimally invasive robotic surgery, vesicourethral anastomotic leakage (VAL) has been reported in approximately 8.0-14.5% of cases [1-3].

Although previous studies have indicated that VAL does not negatively impact continence after RARP [1,3,4], most of these studies focused on cases with minor leakage and without extended catheterization. Moreover, no reports have specifically examined the impact of delayed catheter removal due to VAL on patients’ quality of life (QoL). Thus, the following is the clinical question addressed in this study: How does prolonged urinary catheter retention due to VAL after RARP affect continence recovery and patient QoL?

## Materials and methods

Participants

This retrospective study included 360 patients who underwent transperitoneal RARP at a single center between January 2022 and December 2024. The primary endpoint was the assessment of differences in continence rates between patients requiring prolonged urinary catheterization and those who underwent early catheter removal according to the standard postoperative protocol. Secondary endpoints included differences in QoL and identification of predictive factors for prolonged catheterization due to VAL.

**Figure 1:**
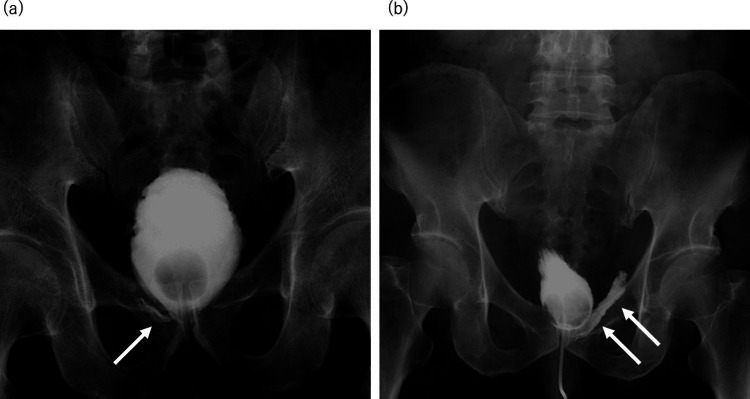
Classification of urinary leakage. (a) Grade 1: linear-shaped leakage. (b) Grade 2: spread strip-shaped leakage

Prolonged catheterization was defined as continued catheter placement beyond postoperative day seven necessitated by VAL. At our institution, all patients began pelvic floor muscle exercises (PFME) on postoperative day two to promote recovery from urinary incontinence [5]; they also underwent cystourethrography on postoperative day six or seven to evaluate anastomotic integrity. The procedure involved the instillation of ~100 mL of contrast medium into the bladder. VAL was graded according to the criteria described by Tohi et al.: Grade 1 - linear leakage without contrast spreading and Grade 2 - spreading, strip-shaped leakage (Figure 1) [3]. If the attending physician determined that prolonged catheterization was necessary, follow-up cystourethrography was scheduled at the physician’s discretion. Although VAL was graded based on cystourethrography findings, the duration of catheterization was not determined according to leakage grade but rather based on overall clinical judgment. Urinary catheter removal in the prolonged catheterization group was performed only after the absence of VAL was confirmed on follow-up cystourethrography; however, the timing of repeat cystourethrography was left to the discretion of the attending physician.

This study was approved by the Ethics Committee of Kansai Medical University Hospital (approval no. 2020215) and was conducted in accordance with the Declaration of Helsinki. The requirement for informed consent was waived due to the retrospective design, although participants were informed of the study and given the opportunity to opt out.

Surgical procedure

All RARP procedures were performed using either the da Vinci Si/Xi® Surgical Robot System® with a six-port technique. Vesicourethral anastomosis was conducted using a continuous suture technique with 3-0 Monocryl by six different surgeons. Posterior and anterior reconstruction was routinely performed [6]. The use of the Retzius-sparing (hood) technique [7] and the advanced reconstruction of vesicourethral support (ARVUS) method [8] was at the discretion of the operating surgeon. Intraoperative leak testing was performed by instilling 100 mL of saline into the bladder before final suturing.

Continence, QoL, and predictive factors

Continence status was assessed using Question 5 of the Expanded Prostate Cancer Index Composite (EPIC) questionnaire: “How many pads or adult diapers per day did you usually use to control leakage during the last 4 weeks?” [9]. Continence was defined as the use of one pad or fewer per day. QoL was evaluated using validated instruments, including the Functional Assessment of Cancer Therapy-General (FACT-G) [10], prostate cancer-specific module (FACT-P) [11], and urinary subscales of the EPIC questionnaire [9]. Questionnaires were administered preoperatively and at 1, 3, 6, 12, and 24 months postoperatively. Risk factors associated with prolonged catheterization were also examined.

Statistical analysis

Continuous variables are presented as mean (range), and categorical variables as number (percentage). The Mann-Whitney U test was used to assess differences between groups. Continence recovery was evaluated using Kaplan-Meier analysis and log-rank tests. Multivariable analysis was performed using logistic regression to identify independent predictors of prolonged catheterization. No missing data were present for variables included in the primary analyses. A p-value < 0.05 was considered statistically significant. All analyses were conducted using Statistical Product and Service Solutions (SPSS, version 28; IBM SPSS Statistics for Windows, Armonk, NY).

## Results

**Figure 2:**
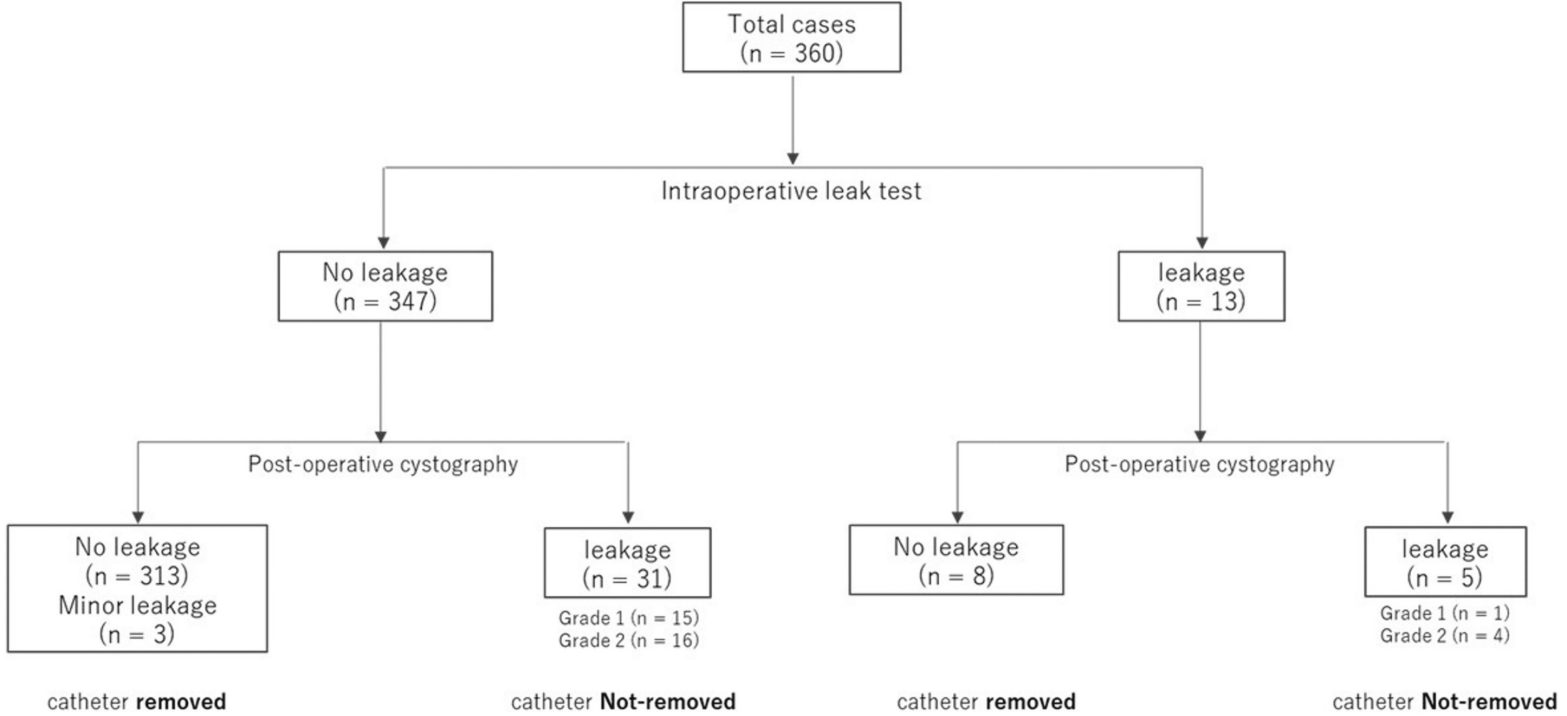
Dendrogram of vesicourethral anastomotic leakage and urethral catheterization during and after robot-assisted laparoscopic prostatectomy (RARP). Overall, 36 of 360 patients (10.0%) required prolonged catheterization after cystography and were classified into the Not-removed group.

Figure 2 presents a dendrogram of VAL occurrence during and after RARP. Intraoperative leakage occurred in 13 patients (3.6%), of whom five (38.4%) showed leakage on postoperative cystography. Four of these five patients (80%) had Grade 2 leakage.

Among 347 patients without intraoperative leakage, 34 (8.9%) exhibited leakage on postoperative cystography. Three patients with minor leakage underwent standard catheter removal. In total, 31 patients required prolonged catheterization, including 15 with Grade 1 leakage and 16 with Grade 2 leakage. Overall, 36 of 360 patients (10.0%) required catheterization beyond the standard period after cystography.

Table 1 summarizes the perioperative patients' data. The Not-removed group had a slightly higher BMI, greater intraoperative blood loss, and heavier prostate specimens. The mean duration of catheterization was six days in the Removed group and 29 days in the Not-removed group.

**Table 1:** Preoperative, intraoperative, and follow-up data for all patients, stratified by the urinary catheter removal status. Values are presented as mean (range) or number (%). Continuous variables were compared between the removed and not-removed groups using the Mann–Whitney U test, and the corresponding U statistics are shown in the Statistical result column. Categorical variables were compared using the chi-square test; for 2 × 2 tables with small expected cell counts, Fisher’s exact test was applied. The corresponding χ² values are shown in the Statistical result column. A p-value < 0.05 was considered statistically significant.

Characteristics	All (n = 360)	Removed (n = 324)	Not-removed (n = 36)	Statistical Result	P value
Age (years), mean (range)	69 (44-80)	69 (44-80）	69 (55-80)	U = 5665.5	0.778
BMI (kg/m^2^), mean (range)	24 (16-38)	24 (16-38)	25 (18-32)	U = 7375.0	0.009
Follow-up period (months), mean (range)	15 (1-34)	16 (1-34)	15 (1-32)	U = 5558.5	0.708
PSA value before surgery (ng/mL), mean (range)	11 (3.0-98)	11 (3.0-98)	10 (4.0-55)	U = 5690.0	0.857
DM, n (%)	46 (12.8)	38 (11.7)	8 (22.2)	χ² = 3.171	0.075
Ischemic heart disease, n (%)	29 (8.1)	26 (8.0)	3 (8.3)	χ² = 0.004	0.953
Neoadjuvant hormone therapy, n (%)	62 (17.2)	58 (17.9)	4 (11.1)	χ² = 1.048	0.307
Biopsy ISUP Grade Group				χ² = 1.562	0.458
Group < 3	138 (38.3)	119 (36.7)	17 (47.2)		
Group 3	76 (21.1)	70 (21.6)	6 (16.7)		
Group > 3	146 (40.6)	135 (41.7)	13 (36.1)		
Initial clinical disease T stage				χ² = 0.607	0.738
cT1	213 (51.2)	191 (59.0)	22 (61.1)		
cT2	134 (37.2)	122 (37.7)	12 (33.3)		
cT3	13 (3.6)	11 (3.4)	2 (5.60		
Console time (min.), mean (range)	199 (93-306)	198 (93-306)	205 (131-300)	U = 6349.5	0.365
Blood loss (mL), mean (range)	339 (0-2171)	327 (0-2171)	450 (50-1818)	U = 7460.0	0.005
Specimen weight (g), mean (range)	41 (12-135)	40 (12-135)	48 (15-113)	U = 7516.0	0.004
Bladder neck reconstruction, n (%)	36 (10)	30 (9.26)	6 (16.7)	χ² = 1.955	0.196
Nerve sparing surgery, n (%)				χ² = 4.390	0.111
None	112 (31.1)	97 (29.9)	15 (41.7)		
Unilateral	217 (60.3)	201 (62.0)	16 (44.4)		
Bilateral	31 (8.6)	26 (8.0)	5 (13.9)		
Intraoperative urinary leakage, n (%)	13 (3.6)	8 (2.47)	5 (13.9)	χ² = 12.139	< 0.001
Postoperative urethral catheterization period, mean (range)	9 (5-60)	6 (5-13)	29 (14-60)	U = 11016.0	< 0.001
BMI, body mass index; ISUP, International Society of Urological Pathology; PSA, prostate specific antigen					

**Figure 3:**
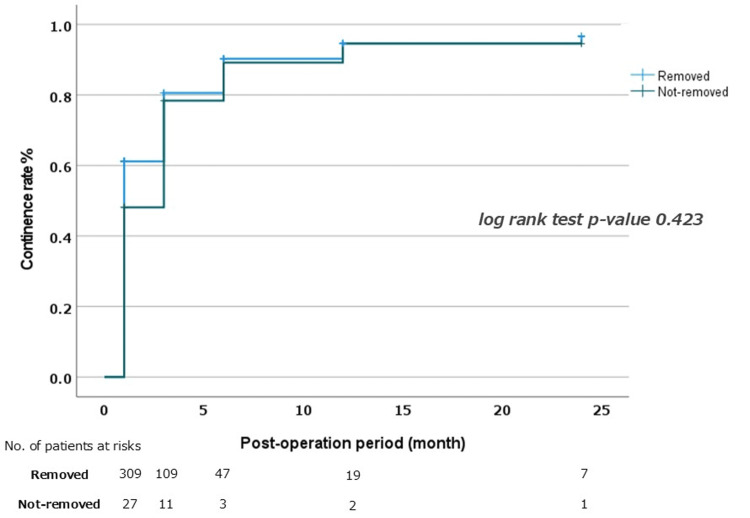
Longitudinal recovery of urinary continence after robot-assisted radical prostatectomy (RARP) according to catheterization duration. Kaplan–Meier curves showing the proportion of patients achieving urinary continence at 1, 6, 12, and 24 months after robot-assisted radical prostatectomy (RARP), stratified by catheterization. Differences between groups were analyzed using the log-rank test, and a p-value <0.05 was considered statistically significant.

Kaplan-Meier curves (Figure 3) showed no significant difference in continence recovery between groups. Continence rates at 1, 6, and 12 months were 63%, 89%, and 92% in the Removed group, and 54%, 85%, and 93% in the Not-removed group, respectively.

**Figure 4:**
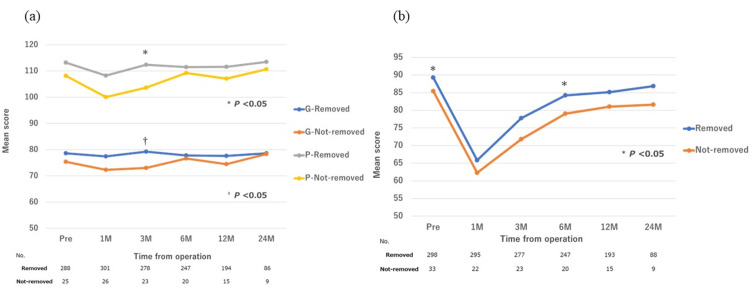
Changes in patient-reported quality of life (QoL) scores according to catheterization. (a) Functional Assessment of Cancer Therapy–General (FACT-G) and prostate cancer–specific module (FACT-P) scores. (b) Expanded Prostate Cancer Index Composite (EPIC) urinary subscale scores. Values are expressed as means ± standard deviation (SD). Comparisons between groups were performed using the Mann–Whitney U test, with p <0.05 considered statistically significant.

QOL outcomes (Figure 4) demonstrated similar trends in FACT-G and FACT-P scores between groups, except at three months postoperatively. EPIC scores were lower in the Not-removed group at baseline and showed a slight divergence at six months.

Multivariate analysis identified prostate specimen weight as an independent predictor of prolonged catheterization (HR: 1.019; 95% CI: 1.000-1.039; p = 0.049). Intraoperative leakage was also strongly associated with prolonged catheterization (HR: 9.506; 95% CI: 2.334-38.725; p = 0.002) (Table 2).

**Table 2:** Risk factors for prolonged catheterization due to anastomotic urinary leakage in patients after robot-assisted radical prostatectomy (RARP). Odds ratios (ORs) and 95% confidence intervals (CIs) are shown. ORs for binary variables represent comparisons between the presence and absence of the condition. Continuous variables were analyzed per unit increase. Multivariable analysis was performed using logistic regression to identify independent predictors of prolonged urinary catheterization. A p-value < 0.05 was considered statistically significant.

Multivariate analysis			
Variables	OR	95％CI	p value
Age	1.008	0.951-1.069	0.777
BMI	1.124	0.995-1.270	0.059
DM (y/n)	1.85	0.728-4.705	0.196
Biopsy Gleason Grade (1, 2, 3)	0.778	0.495-1.225	0.279
Clinical stage (1, 2, 3)	1.478	0.712-3.065	0.294
Console time	0.997	0.998-1.006	0.524
Blood loss	1.001	1.000-1.002	0.109
Specimen weight	1.019	1.000-1.039	0.049
Bladder neck reconstruction (y/n)	1.032	0.329-3.234	0.957
Intraoperative urinary leakage (y/n)	9.506	2.334-38.725	0.002
Nerve sparing surgery (y/n)	0.634	0.332-1.247	0.187
BMI = body mass index; DM = diabetes mellitus; y/n = yes or no			

## Discussion

This study investigated whether prolonged urinary catheter retention due to VAL after RARP impacts continence recovery and QoL. Although 10.0% of patients required prolonged catheterization, there was no significant adverse effect on either continence or QoL. Heavier prostate specimens and intraoperative leakage were associated with an increased likelihood of prolonged catheterization. To the best of our knowledge, this is the first study to focus on the effects of prolonged catheterization resulting from VAL.

Previous research on VAL and urinary continence after RARP has generally concluded that VAL does not negatively affect continence outcomes. Rebuck et al. reported that 27 of 213 patients (12.7%) in their single-center cohort experienced anastomotic leakage; there were no significant differences in continence between patients with and without postoperative urinary leakage [4]. In that study, leakage was assessed based on the presence of urine in daily drain output, and cystography was not performed. The duration of urinary catheterization was also not reported.

Patil et al. conducted cystography in all patients on postoperative day seven and reported a VAL rate of 8.6% in their single-center cohort. They observed continence rates of 70% at three months and 94% at 12 months and concluded that urinary leakage might delay continence recovery. However, they did not report catheter duration or compare outcomes with a no-leakage group [2].

Tohi et al. investigated whether the severity of vesicourethral anastomotic leakage detected by cystography influenced urinary continence recovery. In their retrospective observational study, the continence rate was significantly higher in the leakage group (14.5% of all patients) than in the no-leakage group. The leakage group included patients with minor leakage who underwent catheter removal on the same postoperative day as those in the no-leakage group [3].

To our knowledge, this is the first study to assess whether prolonged urinary catheter retention due to vesicourethral anastomotic leakage after RARP affects continence recovery. Our findings indicate that even extended catheterization does not adversely affect urinary continence outcomes. One possible explanation is that prolonged catheter retention allows sufficient time for anastomotic healing without causing functional impairment of the continence mechanism. As long as structural damage to the sphincter complex does not occur, temporary catheterization itself is unlikely to compromise long-term continence recovery.

In the current era of robotic surgery, most institutions remove the urethral catheter approximately 5-10 days after RARP [12-14], although the duration may vary depending on anastomotic integrity, the presence of leakage, and reconstructive techniques employed. Although Nadu et al. demonstrated the feasibility of early catheter removal on postoperative day two or four after cystography [15], early removal may lead to complications such as pelvic abscess, urinoma, and acute urinary retention [16,17]. These complications may result from minor urine leakage, anastomotic edema, postoperative pain, or increased bladder neck smooth muscle tone [18]. Busby et al. recommended catheter removal on postoperative day seven as a balance between allowing sufficient anastomotic healing and minimizing patient discomfort associated with prolonged catheter use [19]. In our practice, cystography is routinely performed on postoperative day six prior to catheter removal. However, routine cystography is not commonly performed in many centers [4].

The present study showed that prolonged urinary catheter retention did not adversely affect urinary-related QoL. Most QoL scores from the FACT and EPIC questionnaires were consistently lower in the Not-removed group than in the Removed group. The significantly lower preoperative EPIC score in the Not-removed group may reflect baseline differences, possibly due to larger prostate volume, as indicated by the greater specimen weight in this group before surgery.

In multivariate analysis, prostate specimen weight (HR: 1.019; 95% CI: 1.000-1.039) and intraoperative urinary leakage (HR: 9.506; 95% CI: 2.334-38.725) were identified as significant risk factors for prolonged catheterization due to VAL after RARP. These findings suggest that intraoperative leak test results can help predict the feasibility of catheter removal according to the standard postoperative protocol. They emphasize the importance of meticulous vesicourethral anastomosis and offer valuable insights for surgical practice.

This study has several limitations. First, the sample size was relatively small. Second, the retrospective design and absence of long-term follow-up may limit the generalizability of the findings. Third, continence status was evaluated using patient-reported outcomes rather than objective measures such as the pad test. Nonetheless, the study addresses an important clinical question shared by both patients and physicians concerning the effect of urinary catheterization on continence recovery. Further prospective studies with sufficient power and well-defined patient cohorts undergoing standardized surgical procedures are warranted to validate these findings.

## Conclusions

Prolonged urinary catheterization caused by VAL after RARP does not negatively impact long-term urinary continence recovery or patient-reported QoL. These findings provide reassurance to both surgeons and patients that temporary catheter retention, when clinically indicated, is safe and does not compromise functional outcomes.

For patients with larger prostates or intraoperative leakage, careful attention to anastomotic integrity and routine leak testing are particularly important to prevent unnecessary prolongation of catheterization. Future prospective studies are warranted to refine postoperative catheter management protocols and further optimize patient recovery.
